# The relationship between daily difficulties, well-being, and caregiver burden in older adults with cognitive decline

**DOI:** 10.1192/j.eurpsy.2026.10670

**Published:** 2026-07-31

**Authors:** T. Horigome, S. Bun, Y. Yamamoto, F. Nakai, Y. Tazawa, T. Kishimoto, M. Mimura

**Affiliations:** Keio University School of Medicine, Tokyo, Japan

## Abstract

**Introduction:**

Dementia is diagnosed when daily life difficulties become apparent, yet little is known about the specific situations in which these difficulties arise or how they relate to well-being and caregiver burden. To address this, we compiled a list of real-life difficulties from interviews with older adults living with dementia, selected typical episodes, and developed a questionnaire in which participants rated the frequency of experiencing each difficulty.

**Objectives:**

To examine the relationship between daily difficulties in older adults with cognitive decline, their well-being, and caregiver burden.

**Methods:**

This study was approved by the Keio University Ethics Committee, and written informed consent was obtained. Participants met DSM-5 criteria for Major or Mild Neurocognitive Disorder or reported subjective cognitive impairment. The questionnaire covered four domains: mobility (6 items), cooking (10), shopping (6), and housing (18). Responses were rated on a 5-point Likert scale (“1. Always”–”5. Never”), plus “6. Not applicable.” A Gaussian process model was used to estimate latent factors, which were correlated with well-being [Scale of Positive and Negative Experience (SPANE), Flourishing Scale (FS-J), Satisfaction With Life Scale (SWLS)] and caregiver burden [Neuropsychiatric Inventory Questionnaire–Burden (NPI-Burden)]. Questionnaires were completed by participants and, when possible, accompanying caregivers who rated the extent of participant difficulties.

**Results:**

A total of 184 participants were enrolled; after excluding missing data, 171 (75 men, 96 women; mean age 77.08 ±0.51) were analyzed. Mean MMSE score was 23.81 (±0.45). Self-reported data showed significant positive correlations between SPANE-B and difficulties in cooking (r = 0.20, p < 0.01) and housing (r = 0.21, p < 0.01). No associations with NPI-Burden were observed. In caregiver-reported data, SPANE-B correlated negatively with difficulties in mobility (r = -0.23, p < 0.01), cooking (r = -0.20, p < 0.05), and housing (r = -0.18, p < 0.05). FS-J was negatively correlated with difficulties across all domains: mobility (r = -0.25, p < 0.01), cooking (r = -0.23, p < 0.01), shopping (r = -0.20, p < 0.01), and housing (r = -0.25, p < 0.05). NPI-Burden was negatively correlated with caregiver-reported housing-related difficulties (r = -0.17, p < 0.05).

**Conclusions:**

Higher emotional well-being (SPANE-B) was linked to fewer self-reported difficulties in cooking and housing but to greater caregiver-reported difficulties in mobility, cooking, and housing. This discrepancy suggests that high emotional well-being may reflect reduced illness awareness. Higher eudaimonic well-being (FS-J) was associated with more frequent caregiver-reported difficulties across all domains. Caregiver burden was specifically associated with caregiver-reported housing difficulties.

**Disclosure of Interest:**

None Declared

